# Ecological management of cereal stemborers in African smallholder agriculture through behavioural manipulation

**DOI:** 10.1111/een.12216

**Published:** 2015-05-28

**Authors:** CHARLES A. O. MIDEGA, TOBY J. A. BRUCE, JOHN A. PICKETT, ZEYAUR R. KHAN

**Affiliations:** ^1^ International Centre of Insect Physiology and Ecology (*icipe*)NairobiKenya; ^2^ Department of Biological Chemistry and Crop ProtectionRothamsted ResearchHarpendenU.K.; ^3^ Unit for Environmental Sciences and Management, North-West UniversityPotchefstroomSouth Africa

**Keywords:** African agriculture, food security, parasitoids, predators, push‐pull, stemborers

## Abstract

1. Africa faces serious challenges in feeding its rapidly growing human population owing to the poor productivity of maize and sorghum, the most important staple crops for millions of smallholder farmers in the continent, with yields being among the lowest in the world.

2. A complex of lepidopterous stemborers attack cereals in Africa. However, their effective control is difficult, largely as a result of the cryptic and nocturnal habits of moths, and protection provided by host stem for immature pest stages. Moreover, current control measures are uneconomical and impractical for resource‐poor farmers.

3. An ecological approach, based on companion planting, known as ‘push–pull’, provides effective management of these pests, and involves combined use of inter‐ and trap cropping systems where stemborers are attracted and trapped on trap plants with added economic value (‘pull’), and are driven away from the cereal crop by antagonistic intercrops (‘push’).

4. Novel defence strategies inducible by stemborer oviposition have recently been discovered, leading to the attraction of egg and larval parasitoids, in locally adapted maize lines but not in elite hybrids. We also established that landscape complexity did not improve the ecosystem service of biological control, but rather provided a disservice by acting as a ‘source’ of stemborer pests colonising the crop.

5. Here we review and provide new data on the direct and indirect effects of the push–pull approach on stemborers and their natural enemies, including the mechanisms involved, and highlight opportunities for exploiting intrinsic plant defences and natural ecosystem services in pest management in smallholder farming systems in Africa.

## Introduction

Food insecurity continues to bedevil millions of Africa's poor and is likely to worsen with climate change and population growth. The continent has the highest population growth rate in the world, with human population more than tripling in the second half of the 20th century, from 230 to 811 million (FAO, 2011). In spite of this rapid surge in human population, average growth in food production in the continent has at best stagnated (World Bank, 2008). Agriculture is the most important enterprise in Africa (Abate *et al*., 2000), with about 60% of people in the continent earning their livelihood from it (FAO, 2011). It accounts for 35% of sub‐Saharan Africa's (SSA) gross national product, with domestic food production accounting for about 80% of consumption (UNEP, 2002). Growth in agricultural productivity is, therefore, key to economic development in the region.

Grain crops, the bulk of which are produced by smallholder farmers, play a major role in smallholder farmers' livelihoods, with maize, *Zea mays* L., and sorghum, *Sorghum bicolor* (L.) Moench, being the most important food and cash crops for millions of rural farm families in the predominantly mixed crop–livestock farming systems of the region. In spite of the importance of cereal crops in the region, grain yields and land productivity have continued to decline, with yields being generally < 1.0 t ha^−1^, representing some of the lowest in the world (Cairns *et al*., 2013). Consequently, there is a widening gap between food supply and demand, with per capita production steadily declining (World Bank, 2008). Efficient production of cereal crops is, therefore, central to addressing the food security challenge in the region.

## Insect pests as constraints to cereal production in sub‐Saharan Africa

Lepidopterous stemborers constitute one of the major constraints to efficient production of cereal crops in most parts of SSA, with a complex of species attacking these crops (Kfir *et al*., 2002). Of the known stemborer species causing economic yield losses, *Busseola fusca* (Fuller) (Noctuidae) and *Chilo partellus* Swinhoe (Crambidae) are the most important (Kfir *et al*., 2002). While *B. fusca* is indigenous to the region, *C. partellus* was recorded for the first time in Africa in the late 1920s (Tams, 1932), and has now spread throughout eastern, central, and southern parts of the African continent (Harris, 1990). It has become the most important stemborer species at low and mid elevations in East Africa (Van den Berg *et al*., 1991). Stemborer attack results in significant yield losses ranging from 10 to 88% (Kfir *et al*., 2002) of the potential grain output, depending on pest population density and phenological stage of the crop at infestation.

Effective control of stemborers is difficult, largely as a result of the cryptic and nocturnal habits of the adult moths, and the protection provided by the host stem for immature pest stages (Ampofo *et al*., 1986). The main method of stemborer control which is currently recommended to farmers in most of SSA by the public extension agents is the use of chemical pesticides. However, chemical control of stemborers is uneconomical and impractical for many resource‐poor farmers (Van den Berg & Nur, 1998). Moreover, a number of varieties of transgenic maize, with foreign genes from *Bacillus thuringiensis* (Bt) encoding for production of insecticidal crystalline proteins (Lambert *et al.,* 1996), have been developed and implemented in some countries. However, recent field reports indicate that *B. fusca* has developed resistance to Bt‐maize in South Africa (Kruger *et al.,* 2011). Cultural control is the most relevant and economical method available to millions of resource‐poor farmers in SSA (Van den Berg *et al*., 1998), and encompasses a range of activities including destruction of crop residues, manipulation of planting dates, and tillage methods. The majority of smallholder farmers in the region traditionally practice intercropping, a component of companion or multiple cropping, in which they, perhaps unknowingly, manipulate crop microclimates to achieve better crop production (Abate *et al*., 2000). Companion cropping, often with livestock, is a principal means of intensifying crop production both spatially and temporally, and improves returns from limited land holdings, with field studies indicating that the risk to smallholder farmers in such systems is lower than in monocropping (Stigter & Weiss, 1986). In some cereal‐food legume intercropped fields, a reduction in stemborer populations has been observed, although no studies have shown that farmers grow any of the intercrops specifically to exploit this effect (Kfir *et al*., 2002). Moreover, studies designed to establish the underlying mechanisms behind the effect of intercropping on stemborer population levels in such systems are not common.

Although cultural control options are considered cheaper relative to the above, they are not often adopted by resource‐constrained farmers (Chitere & Omolo, 1993), with effectiveness of some of the approaches not empirically demonstrated (Van den Berg *et al*., 1998). Moreover, application of some of the methods is severely constrained by a lack of management capabilities of farmers, especially in areas where farming communities lack support from extension services (Kfir *et al*., 2002). Finding a way to reduce the losses caused by stemborers through improved management strategies could significantly increase cereal production and result in better nutrition and purchasing power for many cereal producers. Therefore, companion cropping involving an intercrop as a low‐input system is potentially of great value in developing world agriculture where chemical inputs are not affordable, and where other forms of low‐input agriculture such as organic farming are practiced (Pickett *et al*., 2010).

## The push–pull companion cropping system

Push–pull is a novel companion cropping system that was developed by the international centre of insect physiology and ecology (*icipe*) in Kenya, in close collaboration with Rothamsted Research in the United Kingdom, and national partners in East Africa with the aim of improving productivity and incomes of smallholder farmers through integrated management of stemborers while addressing other constraints to cereal production in the region. The term ‘push–pull’, first conceived as a strategy for insect pest management by Pyke *et al*. (1987) and later formalised and refined by Miller and Cowles (1990), involves use of behaviour‐modifying stimuli (e.g. semiochemicals) to manipulate the distribution and abundance of a pest and/or beneficial insects for the management of the pest (Cook *et al*., 2007). The push–pull approach described herein involves combined use of inter‐ and trap cropping systems where stemborers are attracted and trapped in a perimeter trap plant (‘pull’), and are driven away from the cereal crop by antagonistic/repellent intercrops (‘push’). It is modelled on the African age‐old practice of growing multiple crops in space and time, and is anchored in the observation that planting different crops together creates more ecological niches for beneficial organisms, such as parasitic wasps or predators, which attack and contain pests (Midega & Khan, 2003). Although more diverse plantings may also offer more niches for pests and diseases too, the likelihood of any one organism breaking out in epidemic levels is greatly reduced.

## Development and effectiveness of the push–pull technology

Cereal stemborers such as *B. fusca* and *C. partellus* are polyphagous and utilise a range of host plants including the numerous grasses that serve as reservoirs during non‐cropping seasons (Khan *et al*., 1997a). *icipe* and partners identified the most attractive and antagonistic plant species for use as trap and repellent intercrops, respectively, from a survey of over 400 grass species in eastern Africa. Napier grass, *Pennisetum purpureum* Schumach, and Sudan grass, *Sorghum sudanense* Stapf, both valuable fodder plants, were preferred to maize for oviposition by gravid stemborer moths, and were subsequently used as trap crops in field trials. Planting Napier grass as a border crop around plots of maize resulted in highly significant reductions in stemborer infestation in maize, with concomitant yield increases of 1–1.5 t ha^−1^ (Khan *et al*., 2000). Although Sudan grass also provided natural control of stemborers by acting as a trap plant and as a reservoir for its natural enemies, it supported high survival of stemborer larvae, with the risk of acting as a ‘nursery’ crop from which pests could multiply and invade the main crop. Its use as a trap plant was, therefore, not encouraged.

During the surveys, molasses grass, *Melinis minutiflora* Beauv, also a valuable fodder crop, was found not to be used for oviposition by stemborer moths. This plant was thus used as a repellent plant in the push–pull strategy after subsequent studies confirmed it emitted repellent semiochemicals (Khan *et al.,* 1997b). Indeed, subsequent field studies demonstrated that its use as an intercrop with maize caused a dramatic reduction (of over 80%) in stemborer infestation (Khan *et al*., 2000). Further efforts led to the discovery that forage legumes in the genus *Desmodium* (commonly known as desmodium) were similarly repellent to stemborer moths and provided effective control of these pests in intercrops with maize (Khan *et al*., 2000). In field trials in western Kenya, it was discovered serendipitously that when maize was intercropped with silverleaf desmodium, *Desmodium uncinatum* Jacq., emergence of a parasitic weed that also represents a serious threat to cereal production in SSA, *Striga hermonthica* (Del.) Benth. (Orobanchaceae), commonly known as striga, was significantly reduced. The combined control of stemborers and striga thus resulted in significant yield increases, from an average of 1–3.5 t ha^−1^, with other *Desmodium* spp. also providing same benefits (Khan *et al*., 2006a), making desmodium the intercrop of choice for the majority of smallholder farmers in eastern Africa where both constraints affect cereal production.

The push–pull approach described herein is so far the most effective and indeed the main push–pull strategy in practice by farmers (Cook *et al*., 2007), and broadly effects management of cereal stemborers through direct and indirect effects, with minimal influence from the surrounding landscape.

### Direct effects on cereal stemborers

Generally, direct defence systems are part of the sophisticated strategies that plants employ to protect themselves from ravages of herbivores and include production of toxins, digestion inhibitors, and semiochemicals repellent to phytophagous insects (Kessler & Baldwin, 2001). Both trap and repellent plants used in the push–pull approach exert direct effects on both adult and immature developmental stages of cereal stemborers.

#### Effects on stemborer moths

**Figure 1:**
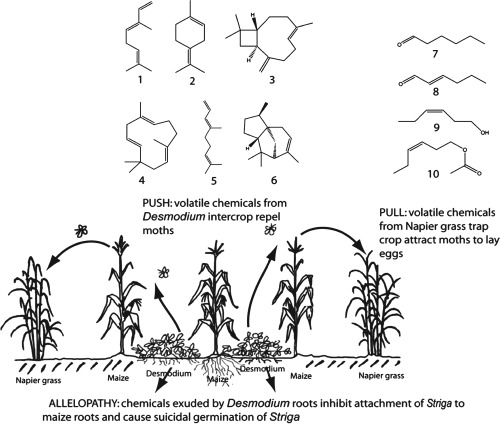
Mechanisms of stemborer control by headspace volatiles emitted by intercrop and trap plants in the push–pull cropping system. Root exudates from the desmodium intercrop cause suicidal germination of striga and inhibit attachment to maize roots. 1 = (E)‐ocimene; 2 = α‐terpinolene; 3 = E‐caryophyllene; 4 = humulene; 5 = (E)‐4,8‐dimethyl‐1,3,7‐nonatriene; 6 = α‐cedrene; 7 = hexanal; 8 = (E)‐2‐hexenal; 9 = (Z)‐3‐hexen‐1‐ol; 10 = (Z)‐3‐hexen‐1‐yl acetate. Adapted with permission from Khan *et al*. (2010).

Through the ‘pull’ effect of the trap plants, the gravid stemborer moths are attracted to and trapped on the perimeter crop. We now know that the attraction of insects to plants and other host organisms involves detection of specific semiochemicals (Dicke & Sabelis, 1988), or specific ratios of semiochemicals (Bruce *et al*., 2005). Analysis of the volatile chemicals from trap plants using gas chromatography coupled–electroantennography (GC–EAG) on the antennae of stemborers led to identification of the key physiologically active compounds responsible for attractiveness of the trap crop to the gravid moths (Khan *et al.,* 2000). These comprised hexanal, (*E*)‐2‐hexenal, (*Z*)‐3‐hexen‐1‐ol, and (*Z*)‐3‐hexen‐1‐yl acetate (Fig. 1). The trap plants released significantly higher amounts of the attractive compounds than maize and sorghum (Birkett *et al*., 2006), with 100‐fold increases within the first hour of scotophase (Chamberlain *et al*., 2006). This coincides with the period during which stemborer moths are most active for oviposition (Päts, 1991). Maize was also found to display a similar response, although the increases were only approximately 10‐fold, accounting for the relative preference of the gravid moths for the trap plants compared to maize.

Studies suggest that avoidance of unsuitable hosts involves detection of specific semiochemicals, or mixtures of semiochemicals, associated with non‐host taxa, with some plants being avoided because they release signals indicating that they are already infested and are, therefore, less suitable as hosts (Pickett *et al*., 2006). Analysis of the volatiles emitted by molasses grass, supported by behavioural studies, revealed that it produced active compounds responsible for its repellence to stemborer moths. These comprised (*E*)‐ocimene, (*E*)‐4,8‐dimethyl‐1,3,7‐nonatriene (DMNT), (*E*)‐caryophyllene, humulene, and α‐terpinolene (Fig. 1). Notably, (*E*)‐ocimene and DMNT belong to a group of semiochemicals referred to as herbivore‐induced plant volatiles (HIPVs) as they are produced during damage to plants by herbivorous insects (Turlings *et al*., 1990, 1995). It was therefore hypothesised, and later confirmed, that these compounds, being associated with a high level of stemborer colonisation and, in some circumstances, acting as foraging cues for parasitoids, would be repellent to ovipositing stemborers (Khan *et al*., 2000). Molasses grass is thus treated as a non‐host because it produces semiochemicals typically emitted by a highly infested maize plant. Desmodium also produced (*E*)‐ocimene and DMNT, together with large amounts of other sesquiterpenes, including α‐cedrene (Khan *et al*., 2000), and effectively repelled stemborer moths and attracted the pest's natural enemies (Midega *et al*., 2009). The combined effects of the trap and repellent intercrops results in significant reductions in stemborer colonisation, translated in reduced damage to the maize (Table 1) and significant improvements in grain yields (Table 2).

**Table 1:** The mean (± SE) seasonal percentage of maize plants damaged by stemborer larvae at 10 weeks after crop emergence in plots of maize planted in sole stands (monocrop) or in push–pull

District	Cropping system	Mean (± SE) % plants damaged by stemborer larvae					
		Long rains 2013			Short rains 2013		
			t‐value	p‐value		t‐value	p‐value
Kuria	Push–pull	3.7 (1.3)	15.0	<0.0001	7.5 (1.6)	4.9	<0.0001
	Monocrop	29.5 (1.0)			16.7 (1.0)		
Migori	Push–pull	10.0 (1.8)	5.02	<0.0001	11.7 (1.5)	3.62	0.0015
	Monocrop	26.2 (2.6)			24.3 (3.1)		
Rongo	Push–pull	8.0 (1.4)	4.36	0.0003	9.7 (1.5)	4.07	0.0005
	Monocrop	22.2 (2.9)			21.0 (2.3)		
Rachuonyo	Push–pull	6.3 (0.9)	6.49	<0.0001	9.5 (1.3)	3.79	0.0010
	Monocrop	20.8 (2.0)			22.7 (3.2)		
Kisii	Push–pull	9.1 (1.2)	4.64	0.0001	8.6 (1.3)	5.13	<0.0001
	Monocrop	22.3 (2.6)			25.1 (2.9)		
Bungoma	Push–pull	14.8 (1.1)	3.15	0.0046	11.7 (1.6)	3.12	0.0050
	Monocrop	23.8 (2.6)			21.8 (2.8)		
Teso	Push–pull	10.9 (1.6)	3.53	0.0019	9.2 (1.4)	4.78	<0.0001
	Monocrop	21.2 (2.4)			20.4 (1.9)		
Bondo	Push–pull	7.3 (1.2)	1.97	0.0614	7.5 (1.4)	4.14	0.0004
	Monocrop	11.1 (1.5)			21.9 (3.1)		
Vihiga	Push–pull	7.6 (1.5)	4.95	<0.0001	9.4 (1.5)	4.61	0.0001
	Monocrop	22.5 (2.6)			23.5 (2.6)		
Busia	Push–pull	7.6 (1.8)	3.52	0.0019	8.5 (1.7)	4.17	0.0004
	Monocrop	19.1 (2.7)			22.7 (2.9)		
Siaya	Push–pull	10.3 (1.9)	4.13	0.0004	10.7 (1.2)	5.35	<0.0001
	Monocrop	22.4 (2.2)			24.6 (2.2)		
Kisumu	Push–pull	8.9 (1.1)	5.81	<0.0001	8.2 (1.3)	3.55	0.0018
	Monocrop	26.5 (2.8)			19.5 (2.9)		

Means represent data averages from 100 maize plants per plot, and from 12 farmers per district. Each farmer planted a push–pull and a maize monocrop plot. Maize (either ‘Nyamula’ or ‘Jowi’) was planted as described in Khan *et al*. (2008b). At 10 weeks after emergence of maize, 100 plants were inspected in each plot for any characteristic foliar damage caused by stemborer larval feeding, and data expressed as percentage of maize plants damaged by stemborers per plot.

**Table 2:** The mean (± SE) grain yields of maize (t ha^−1^) planted in sole stands (monocrop) or in push–pull

District	Cropping system	Mean (± SE) grain yields (t ha^−1^)			
		LR 2013	*t*‐value	SR 2013	*t*‐value
Kuria	Push–pull	3.5 (0.1)	−11.3	3.6 (0.1)	−5.20
	Monocrop	1.6 (0.1)		2.4 (0.2)	
Migori	Push–pull	3.1 (0.1)	−20.7	3.9 (0.2)	−6.93
	Monocrop	1.0 (0.1)		2.0 (0.2)	
Rongo	Push–pull	3.9 (0.2)	−5.42	3.9 (0.1)	−5.43
	Monocrop	2.4 (0.2)		2.4 (0.2)	
Rachuonyo	Push–pull	3.5 (0.2)	−5.55	3.4 (0.3)	−3.40
	Monocrop	2.0 (0.1)		2.1 (0.2)	
Kisii	Push–pull	3.5 (0.2)	−7.90	3.9 (0.1)	−8.29
	Monocrop	1.7 (0.1)		2.2 (0.8)	
Bungoma	Push–pull	5.6 (0.5)	−6.76	3.8 (0.1)	−9.39
	Monocrop	2.1 (0.1)		2.3 (0.1)	
Teso	Push–pull	4.2 (0.1)	−9.84	3.5 (0.1)	−9.30
	Monocrop	2.7 (0.1)		2.5 (0.1)	
Bondo	Push–pull	4.9 (0.2)	−9.66	4.0 (0.1)	−9.12
	Monocrop	2.0 (0.1)		2.1 (0.1)	
Vihiga	Push–pull	6.0 (0.2)	−14.3	5.5 (0.2)	−14.5
	Monocrop	2.1 (0.1)		2.3 (0.1)	
Busia	Push–pull	5.0 (0.3)	−8.16	5.2 (0.2)	−14.8
	Monocrop	2.0 (0.1)		2.1 (0.1)	
Siaya	Push–pull	4.7 (0.4)	−6.20	4.2 (0.3)	−8.79
	Monocrop	1.8 (0.2)		1.6 (0.1)	
Kisumu	Push–pull	4.0 (0.1)	−14.4	3.7 (0.2)	−9.80
	Monocrop	1.5 (0.1)		1.4 (0.1)	

Means represent data averages from 12 farmers per district; all *t*‐values are associated with *P* < 0.0001. At physiological maturity, all maize plants in each plot were harvested and cobs sun‐dried separately for each plot and farmer. The cobs were then shelled and maize grain sun‐dried to 12% moisture content, and weights individually taken for each plot and farmer and data expressed as tones/hectares.

#### Effects on stemborer larvae

Although Napier grass was preferred to maize by gravid moths and received significantly higher oviposition, only minimal larval survival rates accompanied by long developmental durations were recorded on Napier grass (Van den Berg, 2006; Khan *et al*., 2006b). Subsequent studies established that the high mortality of young stemborer larvae was due to a combination of factors. Napier grass has an inherent defence trait that involves the production of sticky sap upon injury inflicted by feeding larvae in an attempt to bore into the stem. This entangles the young larvae and impedes their mobility, resulting in mortality. Additionally, this predisposes the larvae to the activity of the generalist natural enemies such as spiders that are often prevalent in Napier grass fields/strips (Midega *et al*., 2008). Behavioural tests indicate that although gravid moths preferentially oviposit on Napier grass over maize, emerging larvae only feed minimally on the grass, with significant reductions in the amount of food assimilated by the larvae (Midega *et al*., 2011). Moreover, Napier grass has poor nutritional qualities, and this slows development rates of stemborer larvae (Khan *et al*., 2006b), combined with significant reductions in amount of food consumed and assimilated by the larvae.

### Indirect effects on cereal stemborers

#### Effects on stemborer parasitoids

Hymenopterous parasitic wasps play a crucial role in population dynamics of stemborers, and thus attempts have been made to exploit these during the development of the push–pull approach. In attempts to understand why molasses grass was avoided by stemborer moths, we found that although it belonged to the same family as maize and other attractive host plants, it emitted some unique HIPVs including (*E*)‐ocimene and the DMNT, semiochemicals produced during damage to plants by herbivorous insects (Turlings *et al*., 1990, 1995). Indeed a significant increase in parasitism of stemborer larvae by the indigenous parasitoid, *Cotesia sesamiae* (Cameron) (Hymenoptera: Braconidae) was observed in plots intercropped with molasses grass in western Kenya (Khan *et al*., 1997b, 2000). Therefore, while the semiochemicals produced by molasses grass repelled female stemborer moths, they attracted foraging female *C. sesamiae*, with subsequent studies using a Y‐tube olfactometer bioassay confirming that DMNT was responsible for most of this attraction (Khan *et al*., 1997b, 2000). This finding has opened up an opportunity for exploitation of intact plants with an inherent ability to release such stimuli in the development of novel crop protection strategies.

Field studies similarly demonstrated that intercropping maize with desmodium led to significant improvements in stemborer larval and pupal parasitoid activity, with *C. sesamiae* and *Dentichasmias busseolae* Heinrich (Hymenoptera: Ichneumonidae), being recovered from larvae and pupae, respectively, in most of the fields in East Africa (Midega *et al*., 2014a). Results have however been mixed with regards to the activity of egg parasitoids, mainly *Trichogramma* spp. (Hymenoptera: Trichogrammatidae). Studies in Kenya and South Africa have reported a higher number of stemborer eggs parasitised in the maize monocrop than in the maize–desmodium intercrops (Midega *et al*., 2005, 2006, 2009). However, in a recent study we observed that both egg parasitism and larval–pupal parasitism were significantly higher in push–pull than in maize monocrop plots (Midega *et al*., 2014a). In a four‐arm olfactometer study, we observed the attractiveness of *D. uncinatum* flowers to *C. partellus* larval parasitoids (Midega *et al*., 2009). This observation may suggest that most of the attraction of parasitoids to the push–pull plots with desmodium as the intercrop is mediated mostly by the desmodium flowers, and, therefore, these natural enemies are attracted at later crop stages where the most common pest stages would be larvae and pupae.

There are stemborer species that utilise Napier grass as hosts but are not pests of cereal crops, for example, the genus *Poeonoma*, and serve as alternative hosts of parasitic wasps such as *C. sesamiae* (Khan *et al*., 1997a). Minimal survival rates of stemborer larvae on Napier grass is, therefore, favourable for conservation of the parasitoids by providing continuous refuge to natural enemies as well as sources of nectar, pollen, and alternate preys, and provide a further example where increased parasitism of pests on a crop is linked with the presence of both primary and alternate prey.

#### Effects on generalist stemborer predators

Generalist predators play an important role in the regulation of populations of crop pests globally, and may be one of the most important components of integrated pest management in smallholder cropping systems in the developing world (Midega *et al*., 2006) as they come with no additional cost to the resource‐constrained farmers. Cropping systems and associated pest management approaches often influence abundance, diversity and/or activity of such predators. Indeed the relative complexity of such arthropod communities associated with cropping systems is determined by biological, socio‐cultural, and environmental factors, with conventional pest management approaches, particularly the use of broad‐spectrum pesticides, reported to suppress diversity and abundance of a number of generalist predators (Liss *et al*., 1986).

In an attempt to determine the impact of the push–pull approach on the abundance, diversity, and activity of generalist predators, we conducted a series of studies and observed that ants, earwigs, and spiders were often relatively more abundant than the other predatory families that included Coccinellidae, Staphylinidae, Reduviidae, Nabiidae, Chrysopidae, Carabidae, and Gryliidae (Midega & Khan, 2003; Midega *et al*., 2006). The populations of these predator groups were significantly higher in the push–pull than in monocrop plots (Midega & Khan, 2003). Notably, the predators were often recorded in both maize monocrop and push–pull plots but in varying numbers, indicating they were capable of easily traversing the plots, being highly mobile. There were, however, some *Cheilomenes* sp. and *Chrysopa* sp., important stemborer predators, that were only recovered in the push–pull plots (Midega & Khan, 2003).

Ants, that were in all cases among the first colonisers of the fields and represented by several species, including *Crematogaster* spp., *Camponotus* spp., *Dorylus* spp., and *Pheidole* spp., numerically dominated the predator populations, with their populations and diversity (H′) being significantly higher in push–pull than in monocrop plots. Earwigs, on the other hand, were represented by two genera, *Diaperasticus* and *Forficular*, known predators of stemborer life stages (Bonhof *et al*., 1997). From the results obtained thus far, it is indicative that the push–pull approach supports not only a higher population and diversity of these key groups but also enhances evenness in abundance within the plots. This is based on the fact that H′ is defined by both number of species (species richness) and their evenness in abundance (Price, 1975). Indeed in a diverse agroecosystem, a single arthropod species cannot be very dominant, but in a less diverse system, one or two species will be much more abundant than others (Price, 1975).

In subsequent studies, Midega *et al*. (2006) demonstrated enhanced activity of these predator groups in Kenya and South Africa using a combination of natural and artificial infestation procedures. Predation rates of naturally infested stemborer eggs were assessed. Additionally, screenhouse‐reared plants were infested with stemborer life stages in natural enemy exclusion studies. Predation rates of the naturally oviposited *C. partellus* eggs were significantly higher in the push–pull than in the maize monocrop plots. Furthermore, the predation rates of early instar larvae were higher on plants exposed to predators compared to control plants in exclusion cages and substantially higher in the push–pull than in the maize monocrop plots.

Estimates indicate that up to about 90% of *C. partellus* neonate larvae that hatch from eggs disperse through ‘spinning’ or ‘ballooning’ (Berger, 1989). Some of these larvae die during this dispersal, and chances of landing on non‐hosts like the repellent intercrop are high. Moreover, chances of the fallen larvae finding their host are much more reduced in such vegetationally diverse systems, with increased chances of being preyed upon by the enhanced abundance of generalist predators in the system.

From the foregoing, there is a greater potential for stemborer control in the more complex push–pull system and thus support the theory of greater natural enemy density and/or activity in more complex habitats (Root, 1973). Diverse vegetation may provide natural enemies with shelter, food, and alternate prey (Andow, 1991). Colonisation of the diverse system might be due to the greater attractiveness of the polyculture provided by the companion plants in addition to maize, at least at the host habitat–location phase. Alternatively, because colonisation represents not only immigration but also emigration, the greater abundance of the predators in the ‘push‐pull’ systems may have been caused by a more suitable combination of microhabitats in the polyculture, once the habitat was found by these generalist predators (Midega & Khan, 2003; Midega *et al*., 2008). Moreover, this system is associated with reduced soil temperatures and increased relative humidity (Khan *et al*., 2002) thus modifying the microclimate making it more conducive for the generalists. Our studies thus demonstrate the pest management potential of push–pull through enhancement of stemborer predator populations, diversity, and activity.

#### Effects on ground dwelling arthropods

Several groups of ground‐dwelling arthropods are important regulators of ecosystem services in agro‐ecosystems, with the diversity of key species being indicative of the stability, productivity, and complexity of that ecosystem (Tilman *et al*., 1996). As a result of the ubiquitous distribution and functional diversity of such arthropods, conditions of the environment including habitat modifications may have profound impacts on their abundance, diversity, and activity (Midega *et al*., 2008). Using selected groups as indicators, a number of studies indicate that the push–pull approach described within improves abundance and diversity of ground‐dwelling arthropods. In a series of studies, Midega *et al*. (2008) demonstrated that push–pull enhances spider abundance and diversity of key families. These studies used pitfall traps and soil sampling. Numbers generated from pitfall trap catches estimate active density (Southwood, 1978), which is a function of population size, activity, and ease of capture of a species (French *et al*., 2001). By sampling continuously throughout the cropping seasons, we effectively estimated the relative abundance of spiders in both push–pull and maize monocropped plots.

Together with the overall spider populations, members of the Lycosidae were significantly more abundant in the push–pull than in maize monocrop plots (Midega *et al*., 2008). Lycosids have microhabitat preference, with available moisture, leaf litter, and herbaceous vegetation being the cues with which they select microhabitats (Richman, 1995). Moreover, they are frequently encountered in agroecosystems (Van den Berg & Dippenaar‐Schoeman, 1991) and are an important group in the integrated management of crop pests. With spiders being one of the most important predatory groups, and regulators of other ecosystem services in cropping systems, our findings suggest that the abundance of spiders in the push–pull plots should, therefore, be expected to correspond to a high potential for controlling many pest species in the system, and in enhancing ecosystem stability, productivity and ecological health of these cropping systems.

## Impact of landscape complexity

Agricultural landscapes contain diverse communities of generalist and specialist natural enemies that act as regulators of pest populations on crops. Also, a number of pest species are found in these landscapes. While the effect of landscape complexity on natural enemies is well studied, its effect on pests is less known. Stemborer natural enemies are highly mobile (Midega & Khan, 2003), with potential for dispersal among habitats. Their abundance and activity in croplands is, therefore, likely to depend not only on the cropping system but also on the structure of the landscape matrix. The cover of semi‐natural grasslands as a landscape variable has been used as a proxy for landscape complexity and has been shown to correlate positively with natural enemy abundance and diversity (Purtauf *et al*., 2005). However, because some grasses are hosts of stemborers, grasslands may increase colonisation of stemborers in cereal crops, and thus influence the effectiveness of systems such as push–pull. We evaluated the role of landscape composition, using grassland cover as a proxy of landscape complexity, as a spatial effect and cropping systems (push‐pull and monocrop) as a local effect, on stemborer infestation of maize and parasitisation in western Kenya (Midega *et al.,* 2014a).

The main stemborer species encountered was *B. fusca*, with only minimal numbers of *C. partellus*, at < 10%. Although populations of stemborer life stages were significantly lower in maize under push–pull than in the monocrop plots, the effect of push–pull on pest abundance depended on the amount of grassland cover in the surrounding landscape, with the number of stemborer larvae and pupae increasing with increased grassland cover (Midega *et al*., 2014a). *Trichogramma busseolae* parasitised *B. fusca* eggs whereas *C. sesamiae* and *D. busseolae* were recovered from larvae and pupae, respectively. Both egg parasitism and larval–pupal parasitism rates were significantly higher in push–pull than in maize monocrop plots, with a near significant interactive effect of cropping system and grassland cover on egg parasitism, and a trend towards a larger effect of a cropping system in landscapes with low proportional cover of grassland (Midega *et al*., 2014a). However, the extent of grassland cover did not have any significant effects on egg and larval–pupal parasitism. Overall, landscape complexity created by the grassland cover did not improve the ecosystem service of biological control, but rather it provided a disservice by acting as a ‘source’ of stemborer pests colonising the crop.

## Opportunities to further exploit direct and indirect defences through the push–pull approach

### Early herbivory

Insect attack often triggers the production of HIPVs that serve as foraging cues for natural enemies antagonistic to the pests, in what is referred to as indirect defence (Turlings *et al*., 1990). This often occurs as a result of feeding by the larval stages of the pests. Because the natural enemies are attracted as a result of the damage to plants, such biological control approaches are generally not very effective in reducing pest damage in farmers' fields, and, therefore, activity of the natural enemies does not prevent crop yield losses. Defences elicited by the presence of eggs would benefit plants more as they enable defence to be switched on early, before damage is caused to the plant by larvae (Hilker & Meiners, 2006; Bruce *et al*., 2010). Earlier we observed an unusual phenomenon where oviposition by *C. partellus* on signal grass, *Brachiaria brizantha* (Hochst. ex A. Rich.) Stapf., resulted in suppression of the main green leaf volatile (*Z*)‐3‐hexenyl acetate, used in host location by the pest, thereby making the plant ‘invisible’ to ovipositing stemborer females and thus preventing further egg laying by them (Bruce *et al*., 2010). Consequently, the ratio of other compounds relative to (*Z*)‐3‐hexenyl acetate was increased in plants exposed to *C. partellus* oviposition, making the volatile blend more attractive to *C. sesamiae*, than that of plants without oviposition.

Our observation of *B. brizantha* signals suggested there was an opportunity for exploiting early herbivory to enhance pest management. Our follow‐up studies showed that some open pollinated varieties of maize of Latin American origin had increased emission of defence semiochemicals in response to *C. partellus* oviposition, a trait that was not present in standard commercial varieties (Tamiru *et al*., 2011). This increased emission of HIPVs resulted in an attraction of both the egg and larval parasitoids, representing an effective tritrophic response drawing in natural enemies before damage is caused to the crop. Subsequently, we have shown this trait to be present in the locally adapted African open pollinated varieties (OPVs) (Tamiru *et al*., 2012). The majority of smallholder farmers in Africa (about 80%) grow these local varieties (Odendo *et al*., 2001) for their adaptation to local agro‐ecologies, including their resilience to some of the biotic and abiotic stresses, and because they can replant the seeds (Aquino *et al*., 2001). Such ‘smart’ maize cultivars that respond to attack represent an opportunity to make better use of indirect defence traits and, therefore, their use in the push–pull approach not only enhances the stemborer control efficiency of the technology but also improves its ecological effectiveness.

### Plant signalling

**Figure 2:**
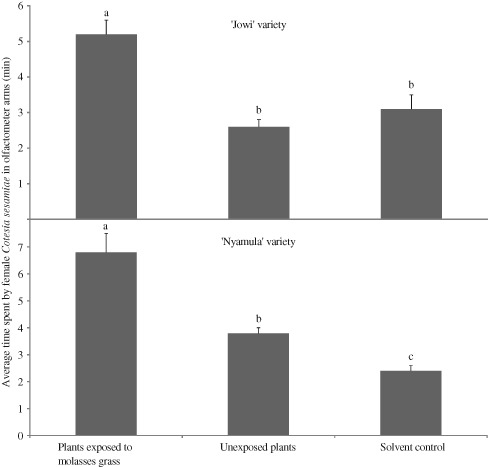
The mean (± SE) time (minutes) spent by female Cotesia sesamiae wasps in the olfactometer arms containing volatiles from maize exposed and unexposed to molasses grass volatiles, and solvent control of the four‐arm olfactometer (n = 30). Averages marked by different letters within a graph are significantly different (P < 0.05).

Plants can respond to HIPVs emitted by neighbouring plants adjusting their metabolism to increase their resistance to herbivores by becoming either ‘repellent’ to the herbivore or more attractive to the natural enemies (Birkett *et al*., 2000). Such plants have a higher expression of resistance genes and defence‐related plant compounds (Arimura *et al*., 2000). We have demonstrated that intact plants such as molasses grass constitutively release similar defence semiochemicals without activity of herbivores (Khan *et al.,* 2000), and can induce defence responses in neighbouring maize plants. Recently, we have observed that local African OPVs ‘Nyamula’ and ‘Jowi’, when exposed to molasses grass volatiles, become significantly attractive to the stemborer larval parasitoid, *C. sesamiae* and less attractive to *C. partellus* moths (Fig. 2). Studies are currently underway to understand the effects, and biochemical pathways involved, of defence inducing volatiles of molasses grass as this will enable exploitation of this trait in development of new plant protection systems based on switching on of inherent plant defences, either through companion cropping or synthetic variants of the active compounds mediating these responses.

## Extending the direct and indirect effects of push‐pull approach to drier areas

Reports indicate that climate change will have far‐reaching effects on cereal production in SSA, posing a further threat to the region's inability to feed its growing population. Recent studies for example, suggest that within two decades, the growing season average temperature will be hotter than any year in historical experience for 4 years out of 10 for the majority of African maize areas, growing to nearly 9 out of 10 by 2050, with rainfall progressively becoming more unpredictable (Burke *et al*., 2009). Incidences of flood and drought, together with atmospheric temperature are also expected to continue to increase, with general increases in the severity of both biotic and abiotic constraints to cereal production resulting in more frequent crop failures. The push–pull approach was developed under optimum rainfall and temperature regimes, and, therefore, not suited to drier and hotter environments. Additionally, it was originally developed for maize but has since been adapted to other cereal crops including sorghum, finger millet (*Eleusine coracana* (L.) Gaertn.), and upland rice (Pickett *et al*., 2010). Therefore to ensure that the approach continues to contribute positively to pest management and farmers' livelihoods, we adapted it to the increasingly drier and hotter conditions associated with climate change. This involved careful selection of drought tolerant companion plants able to deliver the same benefits as the conventional push–pull approach but under harsh environmental conditions.

With funding from the European Union, *icipe* and partners, including Rothamsted Research, identified drought‐tolerant companion plants that deliver similar pest management benefits while providing additional economic benefits. Our studies showed that *Brachiaria* spp. (commonly known as brachiaria), and particularly the commercial hybrid brachiaria cv mulato II grown locally as fodder, could tolerate long droughts of up to 3 months with no water, and more than 30 °C (Z. R. Khan, unpublished). Furthermore, greenleaf desmodium is more drought tolerant, wilts less, and fixes more atmospheric nitrogen than the silverleaf desmodium (Whitney, 1966). Recent studies have demonstrated the beneficial effect of the combined use of brachiaria and greenleaf desmodium in the control of stemborers and striga, resulting in increased grain yields (Khan *et al*., 2014). This ensures long‐term sustainability of the technology and has expanded the geographical appeal into the drier areas of eastern Africa, including Kenya, Uganda, Tanzania, and Ethiopia.

## Uptake of the push–pull pest management approach

Smallholder farmers consider the immediate benefits accruable from any approach aimed at addressing production constraints on their farms, with the majority of them often not considering the long‐term goals (Midega *et al*., 2014b). Economic performance of the push–pull approach has been demonstrated in a number of studies, with the technology out‐performing maize intercropped with edible legumes and maize monocrop (Khan *et al*., 2001, 2008a; Midega *et al*., 2014b). The push–pull approach is highly knowledge‐intensive as its effectiveness depends on proper establishment and management of the companion plants. Supported by social science research on technology transfer, a number of pathways have been used to effectively deliver the technology to smallholder farmers, including storylines on the radio, brochures, and farmers' meetings (barazas). Our earlier studies demonstrated the effectiveness of farmer‐to‐farmer information transfer mechanisms with farmer teachers and field days being some of the most effective (Amudavi *et al*., 2009). We have, therefore, employed these methods to up‐scale the technology to create a nucleus of farmers taking up the technology and thus allowing its horizontal transfer in eastern Africa. By the end of the short rainy season (October–December) of 2014, a total of 96 535 smallholder farmers had adopted the technology in eastern Africa (Murage *et al*., 2015). Although these farmers mentioned effective control of stemborers and improved grain yields after adoption of the technology, a number of other benefits were also reported, including effective control of striga, improved fodder availability, and improved soil fertility (Khan *et al*., 2008b). This confirms the platform nature of the technology that not only provides effective control of insect pests and improves crop yields, but also presents opportunities for integration with other enterprises such as livestock production for overall improvements in farmers' livelihoods.

## Conclusions

This review demonstrates that understanding the interactions of plants with insects and their natural enemies can yield new practical ways of managing crop pests, in this case being delivered by trap cropping and intercropping regimes in a push–pull system. The use of induced defences and plants that produce the desired semiochemicals themselves makes the push–pull approach more sustainable and available, especially for resource‐poor farmers. The approach described within provides effective and sustainable control of stemborers through direct effects on the stemborer moths and larvae, and also through indirect effects via natural enemies, thereby improving the contribution of biological control. Studies are on‐going on the mechanism(s) of natural enemy responses, in both numerical and efficacy dimensions, to the strategy from which a useful generalisation can be made regarding the impact of this kind of companion cropping on stemborer natural enemies. Discovery of useful host defence traits inducible by stemborer oviposition in landraces from South American origin and subsequently in locally adapted African maize OPVs indicates wider occurrence of signaling traits in the crop itself and demonstrates further possibilities for exploiting of this natural plant defense mechanism in African agriculture (Tamiru *et al*., 2012). Moreover, the discovery that this trait appears to have been lost in the elite hybrids paves the way for developing novel and ecologically sound approaches for control of cereal stemborers by (i) introgression of these traits into mainstream commercial hybrid maize varieties (Tamiru *et al*., 2011), (ii) incorporation of these ‘smart’ maize lines into the push–pull approach thereby enhancing its ecological performance, and (iii) enhancing exploitation of the natural process of pest control thereby contributing further to sustainability. The review also demonstrates the value of employing intact plants with an inherent ability for the constitutive emission of beneficial stimuli in the development of effective crop protection approaches.
